# Health-related quality of life of patients diagnosed with COPD in Extremadura, Spain: results from an observational study

**DOI:** 10.1186/s12955-019-1244-4

**Published:** 2019-12-30

**Authors:** María Merino, Renata Villoro, Álvaro Hidalgo-Vega, Concepción Carmona, Collaborative Working Group EPOC-Extremadura, Susana Alonso García de Vinuesa, Anastasia Bejarano Cebrián, Gema Beorlegui Aristu, José Carlos Domínguez Rodríguez, Lorenzo Fernández Prieto, Mª. Carmen Galán Parra, Aurelia María García Martínez, Rosa Mª. García-Adámez Soto, Mª. Mercedes Guío Carretero, Laura Horrillo Murillo, Mª. Teresa Laso Martínez, Laura Martín Jurado, Miguel A. Martín de la Nava, Mª. Eugenia Martínez Domínguez, Carmen Morillo Pantoja, Olga Ortiz Rodríguez, Manuela Pardo Amaya, Ana Mª. Villalba Doblas

**Affiliations:** 1 Fundación Weber, Calle Moreto 17, 5 D, 28014, Madrid, Spain; 2 Universidad de Castilla-La Mancha, Cobertizo de San Pedro Mártir s/n, 45071 Toledo, Spain; 3 Servicio Extremeño de Salud, Avenida de las Américas 2, 06800 Mérida, Badajoz, Spain

**Keywords:** Obstructive pulmonary disease, Health-related quality of life, Primary care, EQ-5D-5L, SGRQ-C, Spain

## Abstract

**Background:**

COPD is a high prevalence chronic disease that involves large reductions of health-related quality of life (HRQL) of patients. This study aims to describe the HRQL of patients with COPD in Extremadura (Spain).

**Methods:**

This is a cross-sectional observational study carried out using a representative sample of patients diagnosed with COPD in Extremadura. The inclusion criteria were patients of legal age, diagnosed with COPD at least 12 months prior to the visit, residing in Extremadura, with electronic medical records available for the 12 months prior to the visit and providing informed consent. The intervention aimed to elicit HRQL indicators obtained from two validated questionnaires: EuroQol - 5 Dimensions - 5 Levels (EQ-5D-5L), and St. George’s Respiratory Questionnaire-COPD (SGRQ-C). The main outcome measures were general HRQL (utility and visual analogue scale) and specific quality of life of COPD patients (total score and three component scores: Symptoms, Activity, and Impacts). Stepwise multiple regression analysis was applied to evaluate the association of EQ-5D-5L and SGRQ-C with respect to clinical and sociodemographic characteristics of the patients.

**Results:**

We recruited 386 patients (mean age 71.8 ± 10.3 years, 76.2% males). In the EQ-5D-5L, participants reported greater problems with respect to mobility (56.5%) and pain/discomfort (48.2%). The mean utility was 0.72 ± 0.31, and the SGRQ-C total score was 40.9 ± 25.0. The results of both questionnaires were associated with number of exacerbations in the last 12 months, level of COPD severity, gender, and education level of the patient (*p* < 0.05).

**Conclusions:**

The results for both utility and total SGRQ-C score indicate that having suffered exacerbations in the last year, presenting a higher level of severity, being a woman, and having a low education level are related to worse HRQL in patients with COPD.

## Background

Chronic obstructive pulmonary disease (COPD) is characterized by an irreversible chronic airflow limitation mainly associated with smoking tobacco [1]. Although there are no recent data on its prevalence in adults in Spain, previous studies estimated the prevalence at approximately 4–10% [2–4]. However, these rates could have been underestimated in some cases, especially when based on the old criteria of the European Respiratory Society for defining COPD. Moreover, it is estimated that 73% of people with COPD are not diagnosed [5]. According to the World Health Organization (WHO), more than 3 million people died in 2015 due to COPD [6], and COPD will be the third cause of death worldwide in 2030 [7]. In Spain, respiratory diseases were the third cause of death in 2015 following diseases of the circulatory system and tumours [8].

As with other highly prevalent chronic diseases, COPD involves an elevated cost, related both to consumption of healthcare resources and to a loss of health-related quality of life (HRQL) [9–13]. In fact, the interest in assessing the HRQL of patients with COPD as an endpoint in itself has grown in recent years [14]. In Spain, studies that estimate the HRQL of patients with COPD outside a hospital setting are scarce. To the best of our knowledge, this is the first study that assesses HRQL and its association with clinical and demographic variables, including COPD severity, using patient-reported-outcome (PRO) measures from a representative sample of COPD patients in one of Spain’s autonomous community (Extremadura). Validated PRO questionnaires provide a standardized method to assess the impact of the disease on patients’ lives [15]. The use of a generic quality of life questionnaire such as the EuroQol-5 Dimensions-5 Levels (EQ-5D-5L), along with the St. George’s Respiratory Questionnaire - COPD (SGRQ-C), allows all aspects related to the health status of patients with COPD to be considered [16].

The primary aim of this study was to describe the HRQL of patients with COPD in Extremadura, one of Spain’s 17 autonomous communities, both in terms of their general quality of life using the EQ-5D-5L, and their specific quality of life using the SGRQ-C. The secondary objective was to explore the relationship between HRQL and several socio-demographic and clinical characteristics of the patients. The data used are part of a study on the total socioeconomic burden of COPD in Extremadura, which describes the economic costs of COPD, and the association between poorer HRQL and increased consumption of health resources [17]. The information provided in this paper complements those results and may prove useful in the design of preventive measures and in decision making related to the appropriate management of COPD.

## Methods

### Subject population

The Extremadura Health Service includes eight different Health Areas. Health Areas are geographical divisions with a relatively decentralized management of primary care services in each area, as is the case across all Spanish autonomous communities. The eight Health Areas of Extremadura are in turn subdivided into a total of 114 Basic Health Areas. According to the Extremadura Health System, there were a total of 9622 people diagnosed with COPD in Extremadura in April 2015 (79.9% males, 79.3% ≥ 65 years), spread across its eight Health Areas. Therefore, our population was 9622 individuals.

### Study design

This study uses data from a larger study dedicated to the socio-economic burden of COPD in Extremadura [17]. This was a cross-sectional observational study that used probabilistic sampling stratified by Health Area in order to obtain a representative sample of patients with COPD in Extremadura. In the first stage, a randomized sample representative of the adult population diagnosed with COPD in Extremadura (*n* = 386, 95% confidence level, 5% sampling error) was calculated based on the prevalence of COPD in the population aged 18 and over in Extremadura. In the second stage, two age group quotas (under 65 years of age and 65 and over) and two gender quotas per age group were established within the sample. These represented the different age and gender prevalence groups in each of the eight Health Areas of Extremadura. We then randomly selected 18 Basic Health Areas distributed across the eight Health Areas of Extremadura, proportionately to the number of Basic Health Areas in each Health Area. The number of patients and age/gender quotas to enrol in each Basic Health Zone were assigned in such a way that the theoretical sample was proportionally distributed across Health Areas. Likewise, within each Health Area, the quotas were proportionally distributed across age and gender of the population with COPD residing in each of the Health Areas.

The present study included people diagnosed with COPD according to the Global Initiative for Chronic Obstructive Lung Disease (GOLD) classification criteria [18] at least 12 months before their inclusion in the study, 18 years of age or older, residents of Extremadura whose primary care electronic medical records were available for the 12 months prior to the collection of the data, who provided informed consent to participate in the study in accordance with local regulations. Patients with any limitation that, according to the researchers’ best judgement, could affect the reliability of their answers were excluded (e.g., patients without knowledge of Spanish, or patients with any evident cognitive impairment). Patients who had participated in any clinical trial in the 12 months prior to the collection of data and pregnant women were also excluded.

A total of 18 previously trained researchers participated in the data collection process, one in each of the 18 Basic Health Areas selected. Researchers received a randomly ordered list of all the patients diagnosed with COPD who resided in their corresponding Basic Health Zone. The patients’ name, contact telephone number, and address were included in the list as they appeared on their clinical record. This information was disclosed to field researchers but was never available to the signing authors of this manuscript. Researchers contacted patients over the telephone following the order in their list. Whenever a patient was not available on the telephone, researchers contacted the next patient on the list. Once telephone contact was established, researchers verified with the patient that inclusion/exclusion criteria were met and, if so, asked the patient to meet in person either in the patient’s home or in their primary care centre, whichever was preferred by the patient. Patients were recruited this way until all age and gender quotas were completed for each Basic Health Zone. The reasons reported by researchers for not enrolling a patient included: the patient does not feel comfortable talking to a doctor, the patient feels nervous and apprehensive about the study, the patient will not be available for a while (e.g. holidays), the patient did not consent to participate, the patient was not reachable after several calls, the patient had a cognitive deficit (Alzheimer’s disease), the patient has moved to another autonomous community or to another Basic Health Area, there was no contact information on the clinical record, and the patient did not recognize having any respiratory disease.

The fieldwork was carried out from July 10th to November 11th, 2015, and included one single visit per patient. Besides collecting information related to healthcare and non-healthcare resource utilization (results published elsewhere [17]), information was collected on patients’ quality of life at the time of the visit using the EQ-5D-5L questionnaire (general quality of life) and the SGRQ-C questionnaire (quality of life of patients with airway obstruction). Information on comorbidities was collected directly from the patients’ electronic clinical records. Prior to data collection, patients were informed about the study objectives and data confidentiality. Patients provided their written informed consent to participate in the study and to release information, according to the Spanish legislation. Permission to perform the study was obtained from the Clinical Research Ethics Committee of the University Hospital Infanta Cristina (Badajoz).

### Study variables

#### Health-related quality of life

Two validated questionnaires were used to assess the HRQL of the patients. The first questionnaire was the EQ-5D-5L, which was used to measure general HRQL at the time of the visit, as reported by the patient. This questionnaire gathers information relative to five health-related dimensions (mobility, self-care, daily activities, pain or discomfort, and anxiety or depression), and each dimension has five response options depending on the intensity level of the problem (no problems, slight problems, moderate problems, severe problems, and extreme problems). Thus, the EQ-5D-5L allows for the collection of a total of 3125 possible health states. Each state is assigned a utility value based on the rates validated for Spain [19], which ranges from 0 (equivalent to death) to 1 (perfect health status). Values below zero are possible and are understood as a health status that is worse than death. This questionnaire further provides a visual analogue scale (VAS) where the patient self-evaluates their health. The VAS ranges from 0 (worst imaginable state) to 100 (best imaginable state) [20].

The second questionnaire was the SGRQ-C, which quantifies the impact of COPD and/or asthma on health and well-being, as perceived by the patients. The questionnaire consists of 50 items divided into three component scores: Symptoms, Activity and Impacts. The Symptoms score refers to the frequency and severity of respiratory symptoms, the Activity score is indicative of dyspnoea-related activity limitations, and the Impacts score shows the psychological and social changes produced by the disease. The items are formulated in two different manners. The first method involves five response options, allowing only a single option to be marked. The second method involves a dichotomous answer: yes/no. A Total score is calculated taking into account all three component scores. All scores range between 0 (without any impact on the quality of life) and 100 (maximum impact on the quality of life) [21]. Researchers provided patients with an electronic tablet containing both questionnaires to be self-administered by the patients.

#### Level of severity

The level of severity according to the GOLD scale (levels I to IV, where I corresponds to mild airflow limitation and IV corresponds to very severe airflow limitation, in patients with FEV_1_/FVC < 0.70) [18] was not available in the primary care clinical records of a large number of patients. As we had no access to clinical records from pulmonology services (these generally include the GOLD classification), we used three complementary sources of information for the elaboration of a single severity variable. Accordingly, the following steps were taken to determine COPD severity: first, the GOLD classification of the patient was used if it was included in the clinical records (10.6% of the patients). Second, in the absence of a GOLD classification, the GOLD classification was calculated from the FEV_1_% predicted values as long as these data were indicated in the clinical records (30.3% of the patients). This calculation was done following the GOLD classification of airflow limitation severity criteria [18]. Third, in cases where no GOLD classification or FEV_1_% data were available, we used the description made by the doctor in the patient’s medical records (0.8% of the patients). Finally, if all the previous data were not available, an “unknown/not available” severity level was assigned. In our sample (*n* = 386), 58.3% of patients (226 patients) lacked information on severity level.

### Statistical analysis

Descriptive statistics (number of valid cases, mean, and standard deviation) were calculated for continuous variables, and frequencies and percentages were calculated for categorical variables. An analysis was performed using the total sample, and comparisons were made based on the level of severity, gender, and age group. For the analysis according to severity, GOLD I and II categories were grouped together, and GOLD III and IV categories were grouped together with the aim of optimizing the statistical comparison. For the comparison of a quantitative variable with a qualitative variable, nonparametric techniques were used (Mann-Whitney U and Kruskal-Wallis U tests). For the comparison of two qualitative variables, the Chi-square test was used. To analyse the explanatory factors of HRQL (utility and SGRQ-C total score), a stepwise multiple regression was used. The regression variables were gender, age in years, education level (no education and primary education vs. secondary and university education), COPD severity level (GOLD I-II vs. GOLD III-IV), number of exacerbations in the last 12 months, and number of comorbidities (this variable includes all comorbidities reported in the patient’s clinical record that were current at the time of the interview). Values of F ≤ 0.05 and F > 0.10 were established as input and output criteria for the explanatory variables, respectively. This process was repeated until the model did not improve. Finally, to correlate the scores of the questionnaires, Spearman’s rho correlation coefficient was used. For all analyses, the limit of statistical significance was *p* < 0.05. The data were analysed with the statistical package IBM SPSS Statistics V22.0.

## Results

A valid sample of 386 patients was obtained. In total, 294 (76.2%) were male. In addition, 306 (79.3%) were 65 years or older with an average age of 71.8 ± 10.3 years old. Table 1 shows the sociodemographic and clinical characteristics of the sample.

**Table 1:** Sociodemographic and clinical characteristics of the sample

Variable	Subcategory	Total
Gender (%)	Male	76.2%
	Female	23.8%
Age in years (mean ± SD)		71.8 ± 10.3
Age group (%)	Under 65	20.7%
	65 years or older	79.3%
Smoking history (%)	Current smoker	16.7%
	Ex-smoker	69.6%
	Never smoked	13.7%
Daily cigarettes (mean ± SD)	Smokers	14.1 ± 10.3
	Ex-smokers	27.3 ± 16.4
Education level (%)	Does not know how to read or write	6.0%
	No education	33.9%
	Primary education	35.9%
	Secondary education	18.5%
	University education	5.7%
Employment status (%)	Early retirement/retired	78.9%
	Actively working	7.9%
	Domestic work	7.1%
	Unemployed	3.4%
	Permanent leave	1.6%
	Temporary leave	1.1%
FEV_1_/FVC (mean ± SD)^a^		60.3 ± 18.7
FEV_1_% (mean ± SD)^b^		66.7 ± 22.3
Level of severity (%)	GOLD I	10.9%
	GOLD II	20.5%
	GOLD III	9.1%
	GOLD IV	1.3%
	Unknown/not available	58.3%
Presence of exacerbations in the last 12 months (%)^c^		36.7%
Number of exacerbations in the last 12 months (mean ± SD)^c^		0.6 ± 1.2
Number of comorbidities (mean ± SD)		7.0 ± 4.0
Main comorbidities (%)	Cardiovascular comorbidity^d^	57.5%
	Dyslipidaemia/Lipid metabolism disorder	41.5%
	Uncomplicated hypertension	38.3%
	Diabetes Mellitus (type 1 + type 2)	27.2%
	Arthritis/Arthrosis	20.0%
	Cancer/malignancy	14.8%
	Hypertension with target organ involvement	13.0%
	Anaemia	6.7%
	Depression	6.2%
	Asthma	6.0%
	Osteoporosis	3.9%
BMI (mean ± SD)^e^		30.0 ± 5.0
Classification of BMI according to the WHO (%)	Insufficient weight	0.8%
	Normal weight	3.4%
	Overweight	16.1%
	Obesity	18.4%
	Unknown/not available	61.4%

*SD* standard deviation, *FEV*_*1*_ forced expiratory volume in the first second, absolute value, *FVC* forced vital capacity, *FEV*_*1*_*%* forced expiratory volume in the first second, percentage value, *COPD* chronic obstructive pulmonary disease, *BMI* Body Mass Index, *MRC* Medical Research Council, *WHO* World Health Organization. Number of valid cases: 386, unless specifically indicated. ^a^Number of valid cases: 123; ^b^Number of valid cases: 140; ^c^Number of valid cases: 215; ^d^Includes unspecified cardiac arrhythmia, atherosclerosis or peripheral arterial disease, valvular heart disease, atrial fibrillation/atrial flutter, acute myocardial infarction, heart failure, cardiac ischaemia with angina, cardiac ischaemia without angina, other cardiac diseases, other cardiovascular diseases, other cardiovascular signs/symptoms, and paroxysmal tachycardia; ^e^Number of valid cases: 149

### EQ-5D-5L: dimensions

**Fig. 1:**
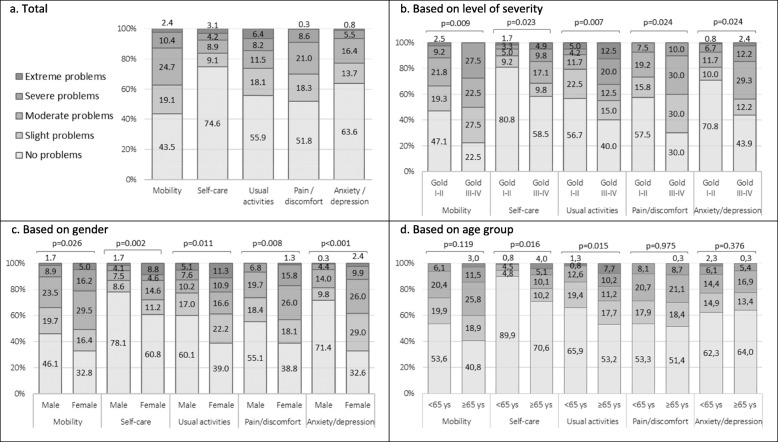
EQ-5D-5L dimensions. Total (**a**), based on level of severity (**b**), based of gender (**c**), and based on age group (**d**)

The health-related dimensions most affected by COPD were Mobility and Pain/Discomfort. The dimension with the highest percentage of severe or extreme problems was Daily Activities. The dimensions with the least associated problems were Self-Care and Anxiety/Depression (Fig. 1a).

All dimensions showed statistically significant differences by severity level of COPD. In general, patients classified as GOLD I-II had fewer problems compared with those classified as GOLD III-IV. These differences were especially evident for the Mobility and Pain/Discomfort dimensions where the proportion of GOLD I-II patients without problems was twice that of GOLD III-IV patients (Fig. 1b).

Furthermore, women endured a higher impact than men in all health-related dimensions. Specifically, in the dimensions of Pain/Discomfort, Self-Care, and Anxiety/Depression, the percentage of women with severe or extreme problems was twice that of men (Fig. 1c).

Moreover, there were statistically significant differences in the Daily Activities and Self-Care dimensions by age group. Patients aged 65 or older reported more severe or extreme problems compared with those younger than 65 years of age (Fig. 1d).

### EQ-5D-5L: utility

The average utility was 0.72 ± 0.31 and varied significantly depending on the level of severity. The post hoc analyses showed no differences between GOLD I and GOLD II categories and between GOLD III and GOLD IV categories. However, differences were found between both groups (GOLD I-II vs GOLD III-IV). The average utility was higher for men than for women, and higher for patients under 65 years of age than for patients 65 years and older (Table 2).

**Table 2:** EQ-5D-5L: Utility and VAS. Total and subgroups (level of severity, gender and age group)

Subgroups	Number of valid cases	Utility		VAS	
		mean ± SD	*p*-value	mean ± SD	*p*-value
Level of severity					
GOLD I	41	0.74 ± 0.35	0.003	59.8 ± 24.4	0.040
GOLD II	79	0.77 ± 0.26		62.4 ± 20.7	
GOLD III	35	0.58 ± 0.31		50.0 ± 21.2	
GOLD IV	5	0.48 ± 0.45		65.7 ± 30.1	
Gender					
Male	308	0.76 ± 0.28	< 0.001	60.5 ± 22.0	0.001
Female	78	0.58 ± 0.38		51.3 ± 20.0	
Age group					
Under 65	80	0.81 ± 0.22	0.023	59.3 ± 20.7	0.837
65 years or older	306	0.70 ± 0.32		58.5 ± 22.3	
Total	386	0.72 ± 0.31		58.6 ± 21.9	

*EQ-5D-5L* EuroQol - 5 dimensions - 5 levels, *VAS* visual analogue scale, *SD* standard deviation, *COPD* chronic obstructive pulmonary disease

### EQ-5D-5L: visual analogue scale

The VAS mean score was 58.6 ± 21.9, but varied significantly by gender, with women reporting a poorer self-assessment of their HRQL than men. Differences were also observed according to level of COPD severity. However, the distribution of utility by severity level was heterogeneous, for differences were only identified between GOLD categories II and III but not among the rest of the groups (Table 2).

### SGRQ-C score

The average total SGRQ-C score was 40.9 ± 25.0 points, with the Activity component having the greatest impact (52.7 ± 28.7 points) on quality of life. The total score showed statistically significant differences by gender and by level of severity. The post hoc analyses did not indicate differences between GOLD I and GOLD II categories nor between GOLD III and GOLD IV categories. However, differences between both groups were found, with greater impact in advanced stages of severity. Women reported a greater average total score than men. The Symptoms component showed a greater impact in advanced stages of severity. The Activity component also revealed greater limitations due to dyspnoea in advanced stages, in women, and in older patients. The Impacts component showed greater psychological and social impact in advanced stages and in women (Table 3).

**Table 3:** SGRQ-C Score. Total and based on subgroups (level of severity, gender and age group)

Subgroups	Number of valid cases	Symptoms		Activity		Impacts		Total score	
		mean ± SD	*p*-value	mean ± SD	*p*-value	mean ± SD	*p*-value	mean ± SD	*p*-value
Level of severity									
GOLD I	41	38.6 ± 22.6	< 0.001	45.7 ± 28.8	< 0.001	27.6 ± 26.8	0.001	35.1 ± 25.1	< 0.001
GOLD II	79	40.3 ± 22.1		53.2 ± 25.9		33.4 ± 26.8		40.7 ± 23.6	
GOLD III	35	62.3 ± 23.0		73.5 ± 23.3		54.8 ± 28.9		61.9 ± 24.8	
GOLD IV	5	55.1 ± 26.1		70.1 ± 25.5		46.3 ± 33.7		55.2 ± 28.2	
Gender									
Male	308	41.6 ± 23.1	0.256	50.6 ± 28.5	0.004	31.1 ± 27.2	0.002	39.0 ± 24.9	0.003
Female	78	44.4 ± 23.3		61.3 ± 28.0		42.0 ± 26.9		48.4 ± 24.3	
Age group									
Under 65	80	39.8 ± 22.2	0.376	44.7 ± 28.7	0.006	28.8 ± 24.7	0.167	35.6 ± 23.4	0.052
65 years or older	306	42.8 ± 23.4		54.8 ± 28.4		34.5 ± 28.1		42.2 ± 25.3	
Total	386	42.2 ± 23.2		52.7 ± 28.7		33.3 ± 27.5		40.9 ± 25.0	

*SGRQ-C* St. George Respiratory Questionnaire – COPD, *COPD* chronic obstructive pulmonary disease, *SD* standard deviation. Columns 3 to 6 show the mean and SD that correspond to the number of valid cases shown on column 2

### Factors associated with HRQL

The results of both regression analyses indicate that having suffered exacerbations in the last year, presenting a higher level of severity, being a woman, and having no education or primary education (as opposed to having a secondary or university education) are related to worse HRQL scores. Patient’s age and number of comorbidities were not statistically significant (Table 4).

**Table 4:** Results of stepwise multiple regression models for utility (EQ-5D-5L) and total score (SGRQ-C)

Dependent variable	Explanatory variables^a^	B coefficient^b^	*p*-value	95% CI	Coefficient of determination
Total score (SGRQ-C)	Intercept	35.604	< 0.001	(29.278; −41.930)	0.431
	Level of severity	21.267	< 0.001	(12.119; 30.416)	
	Number of exacerbations in the past 12 months	8.445	< 0.001	(5.140; 11.749)	
	Education level	−18.147	< 0.001	(−27.469; −8.824)	
	Gender	19.693	< 0.001	(8.864; 30.523)	
Utility (EQ-5D-5L)	Intercept	0.763	< 0.001	(0.684; −0.842)	0.296
	Number of exacerbations in the last 12 months	−0.086	< 0.001	(− 0.127; − 0.044)	
	Level of severity	−0.176	0.003	(−0.291; − 0.061)	
	Education level	0.188	0.002	(0.071; 0.305)	
	Gender	−0.157	0.024	(−0.293; − 0.021)	

*CI* confidence interval, *EQ-5D-5L* EuroQol - 5 dimensions - 5 levels, *SGRQ-C* St. George Respiratory Questionnaire – COPD

^a^Explanatory variables included in the models: gender (0: male (reference category)/1: female), age in years, education level (0: no education and primary education (reference category)/1: secondary and university education), level of severity (0: GOLD I-II (reference category)/1: GOLD III-IV), number of exacerbations in the last 12 months and number of comorbidities

^b^Unstandardized B coefficient

### Correlation between the SGRQ-C and the EQ-5D-5L questionnaires

Utility, as per the EQ-5D-5L, was strongly correlated with the total score of the SGRQ-C (rho = − 0.758). This was higher than the correlation between each of their components separately. On the other hand, the VAS score showed a moderate correlation with the Total SGRQ-C score (rho = − 0.566), as well as with the Activity and the Impacts components, and a low correlation with the Symptoms component.

## Discussion

Few studies have estimated the HRQL of patients with COPD in Spain. To our knowledge, this is the first study to shed light on the relationship between HRQL and several clinical and socio-demographic characteristics of patients with COPD, including severity level, representative of the COPD population in an autonomous community (Extremadura). The main strengths of the present study are the use of a representative sample of adult patients in Extremadura and the use of two different HRQL questionnaires based on PRO measures: a general quality of life questionnaire and a specific quality of life questionnaire.

The total SGRQ-C score of our sample was 40.9 points. This value is similar to that reported by some authors [22, 23] but lower than figures published by others (approximately 44 points) [24, 25]. This difference may be attributed to previous studies using samples with a higher percentage of patients in advanced COPD stages compared with our sample. Pathologies like depression or anxiety significantly influence the total SGRQ-C score [25], contribute to poorer HRQL among these patients [26], and are more often associated with COPD compared with other chronic diseases [27]. In our study, the Anxiety/Depression dimension of the EQ-5D-5L identified a low percentage of patients with some type of problem (*n* = 140, 36.4%). However, the breakdown by level of severity revealed a greater prevalence of Anxiety/Depression in patients classified as GOLD III-IV (*N* = 23, 56.1%) compared with patients classified as GOLD I-II (*N* = 35, 29.8%). Finally, the average utility of our sample was 0.72, which is lower than that reported in previous studies [2, 24, 28]. Therefore, the presence of Anxiety/Depression, advanced stages of disease (GOLD III-IV), and worse health outcomes (reduced utility in EQ-5D-5L and increased Total SGRQ-C score) are interrelated. We believe that our results might reinforce the notion that grouping GOLD cases as I-II and III-IV may be useful and appropriate when analysing the impact of COPD severity on the HRQL of patients [23].

The multivariate analysis revealed that HRQL is influenced by level of COPD severity, number of exacerbations in the last 12 months, education level, and gender. Indeed, we found that advanced stages of COPD and a higher number of exacerbations were related to the worst results in HRQL. These findings are in line with previous studies [29, 30], and underline the importance of developing research on COPD treatments that may hinder exacerbations and lower the progression of the disease. Poor education and being a woman were also associated with worse levels of HRQL. These findings are also in line with previous studies [25, 28, 31, 32], and suggest that any COPD prevention and treatment program should be designed to reach the lower educated segments of the population, and the female population.

Finally, Wacker et al. [24] reported a moderate correlation between the EQ-5D-3 L and the SGRQ-C (− 0.56). The correlation reported between both instruments in the present study is higher (− 0.76), which may be explained by the use of the 5-level EQ-5D instead of the 3-level EQ-5D, providing greater sensitivity in the assessment of HRQL. On the other hand, the same study reported a stronger correlation between the VAS score and the SGRQ-C score (− 0.65) compared to our study (− 0.57).

Of course, this study is not without limitations. First, the primary objective of our observational study was to estimate the socio-economic burden of COPD in Extremadura [17], while measuring the HRQL of patients was a secondary objective. Therefore, given that our sample size was designed to analyse the economic burden of COPD it may not have sufficient statistical power for performing HRQL analyses. Second, the sample design with affixation by gender and age groups could potentially influence the results of the questionnaires, given that these variables influence HRQL. Therefore, populations with different population pyramids and different prevalence rates could yield different results. Third, the severity level of COPD corresponds to a variable constructed from information provided by the clinical history. However, this information was missing for the majority of the participants (58.3%). Indeed, although all patients were diagnosed using spirometry, primary care records only collect information on the number of spirometries that the patient had in the past 12 months, but do not necessarily provide the results of those spirometries, nor the GOLD classification of the patient. Had data been collected from pulmonology services’ clinical records, information on lung function impairment, and other clinical variables such as BMI and number of exacerbations in the past 12 months, would have been available for all patients. Fourth, in our sample, 13.7% of patients never smoked, which is less than that reported in previous studies for Spain [2, 3]. Multiple studies highlight the significant prevalence of COPD among never smokers [33]. However, if we agree that COPD requires an exposure to smoking or to biomass inhalation, then we cannot discard the possibility of an overdiagnosis of COPD in the population of Extremadura, particularly in the elderly population as the FEV_1_% ratio falls with age [2]. Fifth, we have no information on whether particular types of patients were more likely to agree to participate in the study than others. For example, if working patients were systematically more difficult to reach than patients who do not work or who work from home, the sample might suffer from a certain degree of socioeconomic bias. Sixth, the cross-sectional design of our study only allowed us to analyse the association between variables; it did not allow us to evaluate any causal relationship. Finally, our study is only representative of the COPD patient population of Extremadura, one Spanish autonomous community. Extremadura is only one of the 17 autonomous communities in Spain and gathers only 2.35% of the total Spanish population. Although this may limit the generalizability of our results to the rest of the country, the gender, age, socioeconomic distribution, and access to health-care of the population are in line with average values in Spain [34]. Therefore, there is no reason to assume that the HRQL and its association with severity level, gender, and education of the patients differ substantially from other regions in Spain.

## Conclusion

This study sheds new light on the current HRQL of patients with COPD in one Spanish autonomous community (Extremadura). We found that the dimensions of HRQL that were most affected by COPD were mobility, daily activities, and pain or discomfort. We also found that severity of COPD, exacerbations, low education level, and being female are factors that significantly worsen HRQL and each of its dimensions. Health managers should consider these interactions as part of their usual activity in the management of COPD with the ultimate goal of meeting the specific needs of their patients and increasing their HRQL. In particular, any program, strategy, or policy of health promotion regarding COPD should not overlook the importance of preventing exacerbations, where adherence to treatment plays a fundamental role, as well as designing campaigns that target women and less-educated social groups. This study can serve as an example for future HRQL estimations in other autonomous communities or nationwide, with the aim to show the real magnitude of the impact of COPD in Spain. This could help the Spanish National Health System to design, prioritize resources, and improve preventive programmes to enhance HRQL for all COPD patients, in all regions of Spain.

## Data Availability

Data can be shared upon contact with the correspondence author.

## Abbreviations

BMI: Body Mass Index
CI: Confidence Interval
COPD: Chronic Obstructive Pulmonary Disease
EQ-5D-3L: EuroQoL-5 Dimensions-3 Levels
EQ-5D-5L: EuroQoL-5 Dimensions-5 Levels
FEV_1_: Forced Expiratory Volume the First Second, in absolute value
FEV_1_%: Forced Expiratory Volume the First Second, in percentage value
FVC: Forced Vital Capacity
GOLD: Global Initiative for Chronic Obstructive Lung Disease
HRQL: Health-Related Quality of Life
MRC: Medical Research Council
SD: Standard Deviation
SGRQ-C: St. George’s Respiratory Questionnaire
VAS: Visual Analogue Scale
WHO: World Health Organization
